# Hydrodynamic Resistance of Intracranial Flow-Diverter Stents: Measurement Description and Data Evaluation

**DOI:** 10.1007/s13239-019-00445-y

**Published:** 2019-12-03

**Authors:** Benjamin Csippa, Dániel Gyürki, Gábor Závodszky, István Szikora, György Paál

**Affiliations:** 1 Department of Hydrodynamic Systems, Faculty of Mechanical Engineering, Budapest University of Technology and Economics, Budapest, Hungary; 2 Computational Science Institute, University of Amsterdam, Amsterdam, The Netherlands; 3 Department of Neurointerventions, National Institute of Clinical Neurosciences, Budapest, Hungary

**Keywords:** Flow diverter, Stent, Hydrodynamic resistance

## Abstract

**Purpose:**

Intracranial aneurysms are malformations forming bulges on the walls of brain arteries. A flow diverter device is a fine braided wire structure used for the endovascular treatment of brain aneurysms. This work presents a rig and a protocol for the measurement of the hydrodynamic resistance of flow diverter stents. Hydrodynamic resistance is interpreted here as the pressure loss versus volumetric flow rate function through the mesh structure. The difficulty of the measurement is the very low flow rate range and the extreme sensitivity to contamination and disturbances.

**Methods:**

Rigorous attention was paid to reproducibility, hence a strict protocol was designed to ensure controlled circumstances and accuracy. Somewhat unusually, the history of the development of the rig, including the pitfalls was included in the paper. In addition to the hydrodynamic resistance measurements, the geometrical properties—metallic surface area, pore density, deployed and unconstrained length and diameter—of the stent deployment were measured.

**Results:**

Based on our evaluation method a confidence band can be determined for a given deployment scenario. Collectively analysing the hydrodynamic resistance and the geometric indices, a deeper understanding of an implantation can be obtained. Our results suggest that to correctly interpret the hydrodynamic resistance of a scenario, the deployment length has to be considered. To demonstrate the applicability of the measurement, as a pilot study the results of four intracranial flow diverter stents of two types and sizes have been reported in this work. The results of these measurements even on this small sample size provide valuable information on differences between stent types and deployment scenarios.

## Introduction

Intracranial aneurysms (IAs) are localised dilatations or bulges of the arterial wall, most frequently occurring on the Circle of Willis. IAs carry a severe health risk, as rupture leads to subarachnoid haemorrhage (SAH), responsible for approximately 5% of all strokes.^5^ Survivals of the initial effects carry a risk of subsequent complications, which together with the original bleeding, result in a population-based mortality rate of 45%.^44^ In the last few decades, due to the rising use of medical imaging for diagnoses, the detected number of unruptured aneurysms increased.^45^ However, the clinicians still often face the dilemma how to manage such cases: whether to defer or operate.^16^

Conventionally, these lesions were treated by surgical clipping but in the 90’s new endovascular techniques began to gain ground in initial experiments.^2^,^38^ Surgery is complex and often carries increased risk for certain aneurysm locations and sizes. At the beginning of the new century the ISAT, a randomized study comparing endovascular treatment with detachable coils and surgical clipping, as well as its follow-up studies served as the turning point for endovascular techniques (Molyneux^34^; Molyneux *et al*.^35^^–^37). While these techniques used platinum coils to occlude the aneurysm sac, a more recent technology aims at slowing down the flow within the aneurysm sac by flow diverters (FDs) that are densely woven tubular mesh structures deployed in front of the aneurysm neck within the parent artery. These devices were introduced to everyday clinical practice after 2007. In recent years, the use of flow diverter (FD) stents has become an increasingly common endovascular treatment option.^17^,^18^,^26^,^27^,^42^,^46^ Just like coil packing or any other endovascular method, this therapy is minimally-invasive, and post-interventional recovery time is much shorter. The risks of complications must be carefully and individually weighed against the risks of a future spontaneous aneurysm rupture to offer the best care to each patient.^50^ Despite its growing popularity, FD stenting still has weaknesses. Clinical case studies reported continued aneurysm growth and subsequent rupture^14^,^29^ following seemingly successful FD treatment. To understand reasons for delayed aneurysm ruptures following FD deployment, the effect of the stent on the hemodynamic properties has been widely studied in Kulcsár *et al*.^13^,^28^

Although the long term efficacy of FD in terms of permanent aneurysm occlusion has been proved by the 5 years follow up data in the PUFS study,^6^ some aneurysms fail to occlude. There are numerous clinical follow-up and retrospective studies on occlusion rate but the deployment properties of stents are rarely analysed.^1^,^22^*In*-*vitro* experiments are particle image velocimetry (PIV) measurements,^10^^–^12,15 mostly to validate Computational Fluid Dynamics (CFD) results^9^ or 4D MRI results.^7^,^20^ CFD is especially suited for research because it allows the analysis of a variety of FD scenarios and vasculature configurations.^4^,^24^,^31^,^32^ Most of these investigations directly model the micro-structure of the stent, though simplifications are necessary. These include: modelling blood as a Newtonian fluid, taking into account only the ostium for the stent—which assumes that stent apposition on the arterial wall is ideal and does not modify the hydrodynamics locally—and using rigid and hydraulically smooth walls.^3^,^41^ Despite numerous simplifications, the complexity of the model, which requires partitioning space and time in small intervals, remains high, translating into long computational times that often require specialized computational equipment and are not compatible with real-time analysis in every day medical practice.^3^,^24^ The segmentation and smoothing methods require skills and experience, and make the simulation process even more time-consuming and difficult to reproduce. Furthermore, due to the quasi-random nature of the implantation and some imaging limitations, the real configuration of an implanted FD, e.g. the location of individual struts, is not known. This is the result of the woven structure, in which the struts can bend and slide on top of each other, depending on the local vessel diameter, curvature, and implantation process. It is also possible to model the stent as a homogeneous porous layer.^8^,^52^ In this case, meshing becomes simpler, fewer elements can be used, and the uncertainties regarding the exact position of the stent wires are not apparent. Simulation and pre-processing time can be greatly reduced. Including this idea into a simulation package can lead to a very fast, off-the-shelf method that can help medical practitioners in predicting the effectiveness of a planned treatment in a clinically relevant time frame.

In order to model the stent as a porous layer with homogeneous permeability, reliable measurement results on the resistance parameters are needed. In the literature, two parameters, Metal Surface Area (MSA) and pore density (PD) have been used to characterize individual devices. The first systematic studies of the aforementioned parameters were performed by Shapiro *et al*.^39^,^40^

Mechanistically, FDs act as a porous interface between the aneurysmal cavity and its parent vessel and favourable outcomes are achieved, when the flow through this barrier is reduced enough—in others terms reaching sufficient hydrodynamic resistance—to induce stasis, the coagulation cascade and ultimately the scarring of sac. Therefore, the only relevant parameter of the flow limiting capacity of a FD deployment scenario is the hydrodynamic resistance. Additionally, it has to be mentioned that the efficacy of a FD implantation relies on other factors as well, such as remodeling of endothelial cells and activation of platelets inside the aneurysm. To the best knowledge of the authors, no attempt was made to measure this in a comprehensive way. Some early works in this direction will be cited later in this paper.

Resistance of mesh-like structures was measured in other research fields like petroleum science, where they are used for permeability measurements.^30^,^33^,^51^ The methods used for analysing the results are more reminiscent of those used in filtration technology, where Darcy’s law is widely applied.^47^^–^49

The pressure-drop caused by a hydrodynamic resistance (HR) can be characterized by a linear and quadratic term, as a function of flow rate. We decided to describe HR in this paper by the coefficients of these two terms. In our opinion it brings no advantage to use the non-dimensional form of the variables here, since the rig-specific resistance values are more suited to demonstrate the feasibility of the test rig.

Ultimately, our goal is to use our measurements to guide the design of FDs and pre-interventional planning as clinicians commonly struggle to determine the optimum clinical option, such as the pattern, size and position of FDs for specific vascular anatomies. A secondary objective is to look for the relationship between the HR and the above-mentioned geometrical parameters. These data can help determine the effect of various configurations (i.e. various stent types or oversizing), as well provide input data for numerical flow simulations. By knowing the hydrodynamic effect of these parameters, the impact could be quantified and could help in device planning for the clinicians.

## Methods

### General Considerations

**Figure 1:**
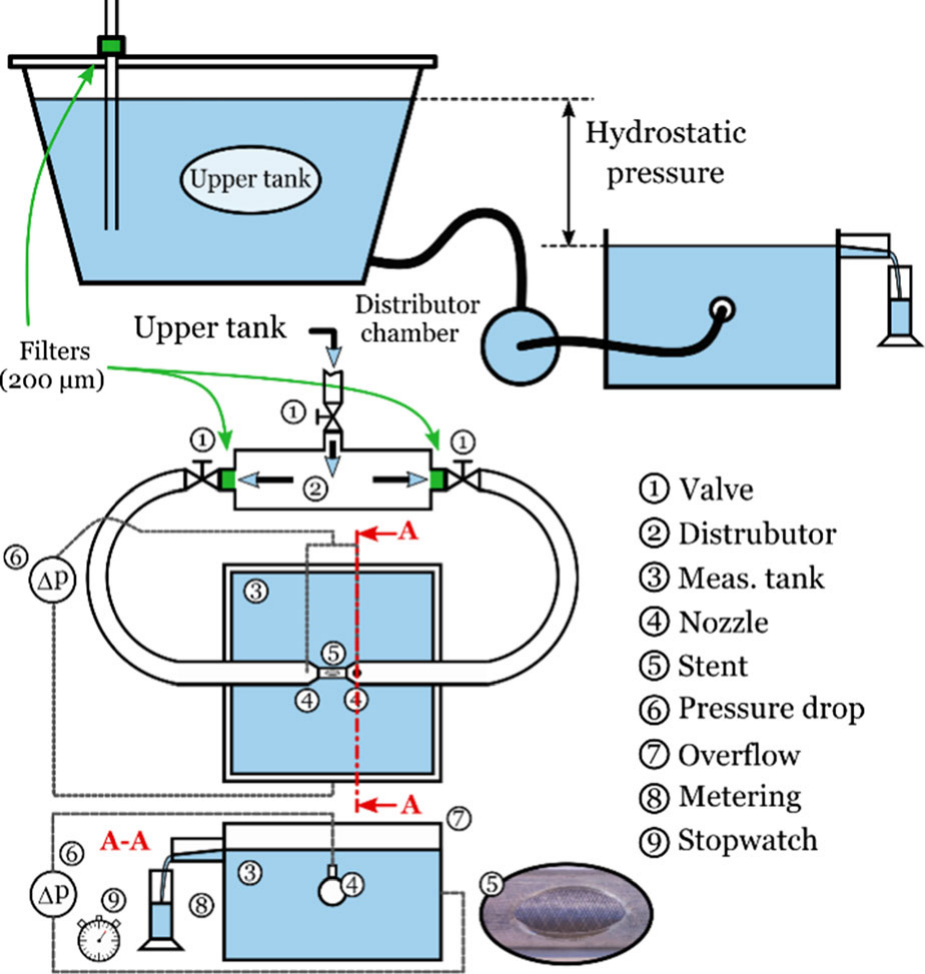
Sketch of the measurement rig layout (final design).

The aim of the measurements is to determine the pressure drop on the FD stent as a function of the flow rate in different configurations. Water was used for the measurements as we assume the blood to behave as a Newtonian fluid. Hence after the measurements the volumetric flow rate measured with water could be converted to blood flow rate by utilising Reynolds number similarity. The sketch of the experimental rig is shown in Fig. 1. The stent is placed into a holder tube (5) with an elliptical opening in the middle that resembles the neck opening of the aneurysm. The stents are implanted into the holder tubes by a neurointerventionist to ensure the similarity and variability of a real world scenario. To provide controlled conditions corresponding to the Darcy–Forcheimer equation, the outflow is perpendicular to the stent surface achieved by a symmetrical layout. Hence, water enters the holder tube from both sides through the nozzles (4). The pressure is measured upstream of the holder tubes, and in the tank, at the height of the outflow.

The flow is driven by hydrostatic pressure by placing a second tank (upper tank in Fig. 1) in a higher position. The flow rate is controlled by letting the water level change in this tank. Such a solution is practical for two reasons. First, a pump would produce unwanted disturbances in the inflow. Second, setting a measurement point would be uncertain, since fluid speeds are very low, (typically between 0.1 m/s and 0.003 m/s) and flow control valves work accurately only when the speed is significantly larger. Instead, lowering the water level in a tank accurately sets any desired working point.

After exiting the modelled aneurysm neck opening (5), the water flows through the stent surface into the outflow tank (3), the surface of which is open to ambient pressure. The water level in the tank cannot rise, since the surplus fluid departs through an overflow (7), and thus the volumetric flow rate can be measured by metering. It is possible to model different diameter artery sections by changing the holder tubes and the upstream sections of the system. The rig is very sensitive to contamination, and so filters are used to prevent contaminants from getting into the system (marked with green in Fig. 1). Air bubbles or solid particles would eventually be caught on the fine mesh of the stent, and would cause severe measurement errors.

Naturally, it is not only the resistance of the stent that we measure but also the pressure drop on the simplified artery model and the outflow. To determine the resistance of the stent alone, two sets of measurements are carried out for each measured stent. First a measurement with the FD stent takes place—$\Delta p_{\text{full}} \left( Q \right)$—followed by an “empty” measurement to get the pressure drop as a function of the volumetric flow rate for the empty system alone $\left( {\Delta p_{\text{empty}} \left( Q \right)} \right)$. The description of the subsequent evaluation procedure can be found in “Measurement Protocol” section. Until now two complete systems were build. The following subsection will briefly summarize this iterative design process that led to the final design.

### Measurement Rig Design Process

**Figure 2:**
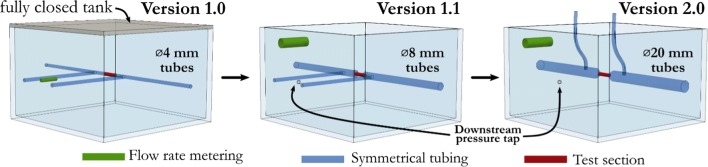
Measurement layout changes. The colours represent the main parts that had been changed during the design development.

**Figure 3:**
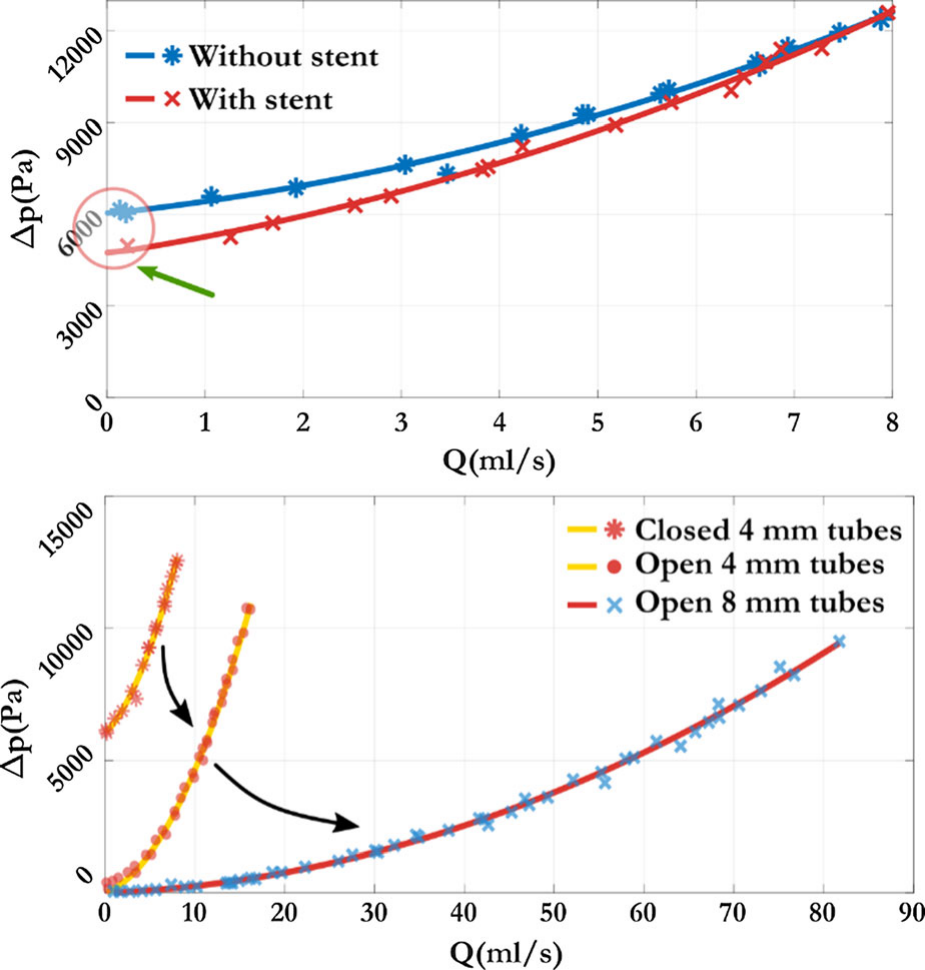
Left: The recurring issue with the closed tank concept. Right: The effect of major design changes on the $\Delta p\left( Q \right)$ curves.

The measurement principle described in the previous section satisfies our requirements. The sketch in Fig. 1 depicts the last generation device which is a refined new version of our second device. Conceptually the first design consisted almost the same elements as in Ugron *et al*.^43^ A symmetrical layout with perpendicular outflow in a tank where the flow rate was provided by the hydrostatic pressure. However, the mechanical parts the system consisted of were completely different. Figure 2 shows and Table 1 lists the main changes. One of the main differences compared to the other layouts was the closed tank concept. Here the upper tank was located 6 m above the lower one and the flow rate was adjusted by a ball valve. The system was assembled from Ø4 mm pneumatic tubes (blue in Fig. 2) and brass T-junctions both before the test section (red in Fig. 2) for the upstream pressure taps and as the flow divider before the symmetric layout. A differential manometer was used to measure the pressure drop. The averaged upstream pressure, and the ambient pressure were connected. By knowing the hydrostatic pressure in the closed tank we could calculate the actual pressure drop on the test section. Since the tank was closed during the measurement, the metering location (a small flexible hose depicted with green in Fig. 2) was beside the two upstream pressure taps. The main difficulty was to completely de-air the tank. After the tank was sealed, other problems occurred: the dissolved gas content in the water often formed new bubbles that introduced unacceptable errors into the measurement. Another source of error was the movement of the metering pipe. Often the small flexible hose deformed, making the flow rate measurement erroneous. As a results of these flaws in the system, the measurements were often inconsistent and hard to reproduce. On the left hand side in Fig. 3, the accumulation of such errors is shown that occasionally lead to the physically impossible result that the pressure drop with the FD stent implanted in the system was smaller than without.

**Table 1:** Main differences between measurement layouts.

	Version 1.0	Version 1.1	Version 2.0
Tank	Watertight closed	Open design	Mechanically closed
Tubes	4 mm pneumatic tubes	8 mm pneumatic tubes	Number
Metering	Small flexible hose	At overflow	At overflow
Pressure measurement	Pressure sensors	Pressure sensor	Inverted U-type manometer
Pipe fittings (“nozzle”)	Brass stock fittings	Brass stock fittings	Specifically manufactured
Fluid distribution	Brass T-junction	Brass T-junction	Distributor chamber

In order to overcome these problems, we abandoned the closed tank concept and only a simple cover was used to exclude the possibility of contamination. The downstream pressure measurement changed accordingly to a direct method as the pressure tap was moved to the side of the tank at the same height as the upstream pressure taps. This way the differential manometer measures the pressure drop directly. Furthermore, we replaced the Ø4 mm pneumatic tubes before the test section to Ø8 mm ones, to decrease the resistance of the empty system. The consequence of the changes can be seen in Fig. 3 on the right. Just by changing the pipe diameter, the pressure loss was reduced by one order of magnitude. The problem caused by the dissolved air inside the tank completely vanished due to the open tank concept. Furthermore, the overflow tube was replaced by a stronger hose of larger diameter. Hence the flow rate became stable even under the highest flow rates since the tube did not deform during the measurements.

Although the measurement built with Ø8 mm pneumatic tubes can perform well, this system also had a number of serious drawbacks. First, the test section is only a Ø4 mm pneumatic tube with a hole cut out by hand, which is not perfectly reproducible if a replacement is needed. Second, the cast stock brass T-junctions serve as sudden flow constrictors before the test section which introduce an inherent instability into the measurements. Thus we came to a conclusion that based on the gathered experience about the requirements and uncertainties, we had to completely redesign the system and manufacture a new test rig.

The new test rig (see sketch in Fig. 1) was designed around the concept that flow disturbances should be kept at minimum before the upstream pressure taps and flow resistance should be as low as possible. First, we abandoned the idea that the operation point is set by the ball valve. The reason is that any kind of valve can cause disturbances in the system, and such valves are not suitable to set flow rates under 10 ml/s. Therefore, the upstream pressure was adjusted purely by the hydrostatic pressure, thus by the level of the fluid in the upper tank. From the entry to the nozzles, the measurement rig is designed to have the lowest possible flow speeds to be in the physiologically relevant Reynolds number range. The larger distributor chamber was designed (formerly a simple brass T-junction) to even out the inflow, and to direct the water towards the branches in the symmetric layout. (We remark here that the valves are not used for flow control, only to fully open or fully close the branches.) Leading from the distributor chamber towards the nozzles, the diameter of the flexible tubes (transparent silicon tubes) and the brass tubes was Ø20 mm. Since the friction resistance is inversely proportional to the fifth power of the inner diameter of the tube, the resistance of the upstream system reduced even more compared to that with the Ø8 mm tubes. Changing the nozzles is quick from one artificial artery size to another one but attention has to be paid to completely de-air the system. To further improve the water quality, we inserted 200 *µ*m filters in the system right after the flow distributor and another one to clean out the tap water entering into the upper tank. The confuser nozzles are specifically manufactured for each artery size, so that disturbances could vanish entirely before the test section.

The key components of managing the reliability of this measurement are the nozzles and the test section (No. 4 and 5 in Fig. 1, respectively). These parts underwent several alterations, and since this environment has the most significant effect on the outcome of the measurements, rigorous attention had been paid to their development. At first, we manufactured the nozzles to have a conical rib at the end so that we can pull on flexible PVC holder tubes. The idea was that the PVC tubes are flexible enough that we could adjust their position easily and as they are fully transparent, we can determine the success of the hand deployment of the FD. However, based on the initial measurements, reproducibility of the measurements was unsatisfactory. Small deflections and marginal differences resulted in an unacceptable change in the pressure drop of the empty system. The cutout (the aneurysm neck) was made by hand, thus it could not be guaranteed a similar behaviour of different PVC tubes, because of poor manufacturing reproducibility.

**Figure 4:**
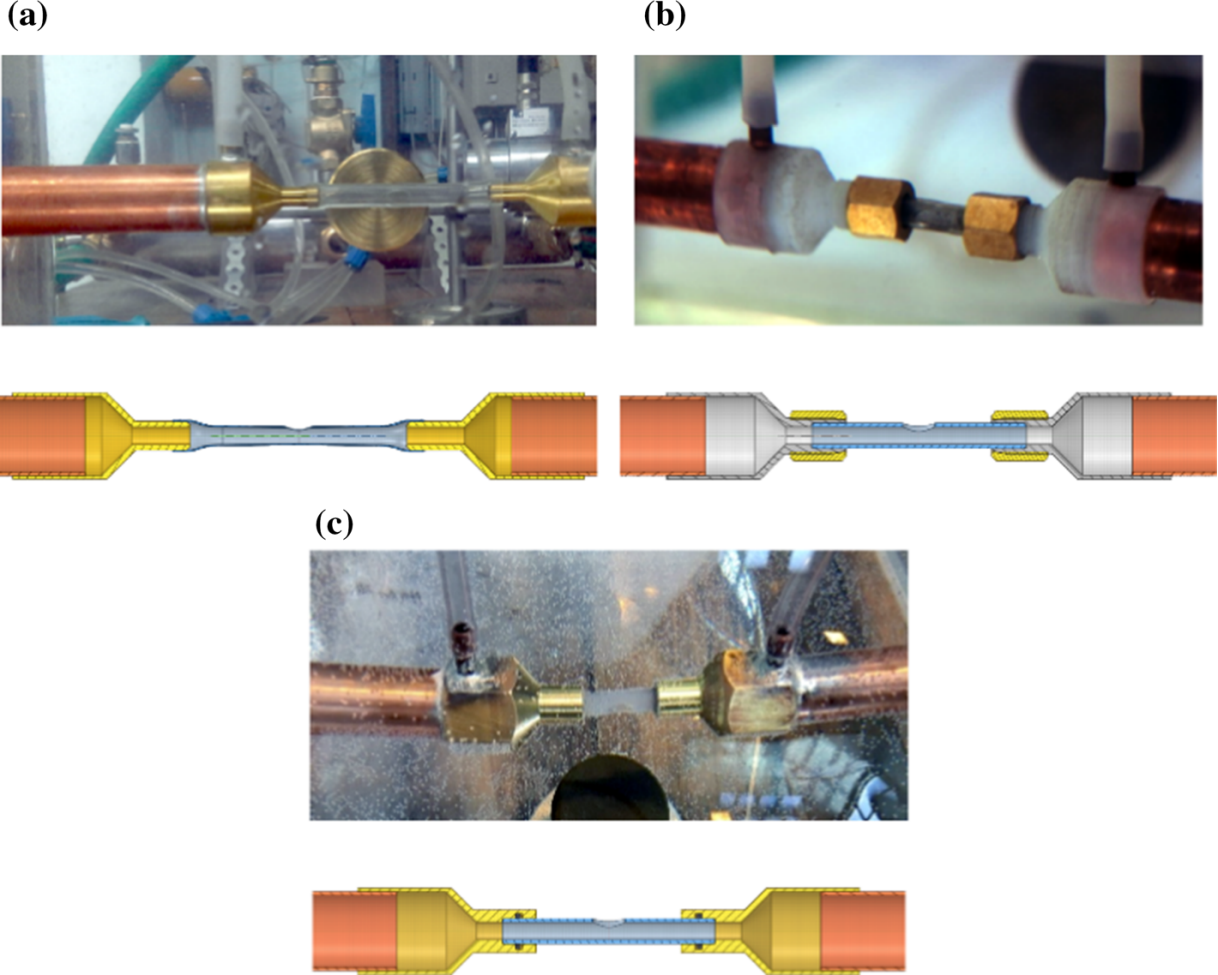
Development stages on the nozzle and the test section in the second measurement rig.

The necessary manufacturing quality was provided by redesigning of the nozzle and the use of 3D printing. Figure 4b in the upper row depicts the developed nozzle-holder tube assembly. As the figure demonstrates, flow deflections are completely eliminated with this configuration, which then provides adequate conditions for the hydrodynamic resistance measurements. For more than a year we used this configuration and the reproducibility of the results improved significantly, though some issues with the 3D printed tubes emerged. Although these problems did not manifest in measurement inaccuracies, they made the measurement procedure slow and cumbersome. These included:

- sometimes the holder tube got stuck in the nozzle and it could not be removed without breaking it;
- having spent some time in the water the tubes became softer but after drying they warped and the previous point happened;
- after a few months the tubes became so brittle that they snapped between our fingers.

Whichever of the above events occurred, it caused delays in the measurements and as the material deteriorated with time we could not build a stock. Consequently, from a certain point we started to manufacture the tubes out of plexiglass with CNC milling and turning, and we even modified the nozzle to ease up the change between measurements. The fully transparent plexiglass holder tubes alleviate the insertion procedure. This second generation nozzle can be seen at the bottom in Fig. 4. In addition to the improved robustness, the surface of the tube is significantly smoother, almost polished, and so the tubes are easier to handle, thus the reproducibility of the measurements improved further.

### Measurement Protocol

In order to ensure controlled measurement circumstances in this study, a measurement protocol was laid down. After implantation of the FDs into the holder tubes by a medical expert (I.Sz.) the testing procedure starts with a microscopic photograph (USB microscope, 20 × magnification, 30-megapixel resolution) for further image processing. Then the implanted stent length is measured before placing the holder tube into the measurement device. Prior to the measurement the old filters are replaced by a new clean one to prevent contaminants from entering the test section. Four consecutive measurements are made—four $\Delta p_{\text{full}} \left( Q \right)$ curves—with an image taken by the USB microscope before each individual measurement. After the four measurements we remove the stent from the holder tube and replace the mechanical filters in the system, then we repeat the hydrodynamic resistance measurement four times to obtain the $\Delta p_{\text{empty}} \left( Q \right)$ curves. Finally, the unconstrained length and diameter of the FD is measured and another microscopic image is taken.

### Data Evaluation

The following part of this paper describes the data evaluation in a greater detail. Based on the Reynolds number similarity, the flow rate measured with water can be converted to blood flow rate as follows. The Reynolds number is the most commonly used non-dimensional number of fluid mechanics; it represents the ratio of inertial and viscous forces.

1 $$Re = \frac{\rho vd}{\mu },$$

where *v* is the cross sectional average velocity, ρ is the fluid density, *µ* is the dynamic viscosity and d is the characteristic length of the flow configuration. Equation (1) can be rewritten as:

2 $$Re = \frac{\rho Qd}{\mu A},$$

where *Q* and *A* is the flow rate and some cross section, respectively. The basis of flow modelling is the equality of the non-dimensional numbers in the “real” and the “model” configuration. We do not have to specify A and d, as long as they are the same in the “real” and the “model” configuration. Here our model is:

3 $$Re_{\text{Water}} = Re_{\text{Blood}} .$$

Thus after rearrangement Eq. (3) simplifies to:

4 $$Q_{\text{blood}} = \frac{{\mu_{\text{blood}} \rho_{\text{water}} }}{{\mu_{\text{water}} \rho_{\text{blood}} }}Q_{\text{water}} = C \times Q_{\text{water}} ,$$

where *µ*_blood_ and *µ*_water_ are the viscosity, *ρ*_blood_ and *ρ*_water_ are the density of the blood and water, respectively. Since the material properties of blood are known, the value of C only depends on the water temperature used during the measurement. Considering a blood density and viscosity of 1055 kg/m^3^ and 3.4 mPa s, respectively, Table 2 shows the dependence on the water temperature. Since tap water was used for the measurements, the temperature of the water had to be recorded every time.

**Table 2:** Temperature dependence of C.

Water temperature [°C]	*C*
10	2.46
15	2.83
20	3.21
25	3.61

Blood density and viscosity were assumed to be 1055 kg/m^3^ and 3.4 mPas respectively

#### Interpretation of a Deployment Scenario

From here onwards flow rate will mean blood flow rate after the conversion. After the measurements of one scenario and conversion of the data, a polynomial curve can be fitted first to the empty point pairs {$\Delta p_{\text{empty}} ;Q_{\text{empty}}$}:

5 $$\Delta p_{\text{empty}} = a_{\text{empty}} Q^{2} + b_{\text{empty}} Q$$

In Eq. (5) above, the quadratic—$a_{\text{empty}}$—and linear—$b_{\text{empty}}$—coefficients are well reproducible. The standard deviation of the coefficients is two orders of magnitude smaller than their mean value after four consecutive measurement series. Using the empty system coefficients we can subtract a corresponding pressure drop from the measured $\Delta p_{{i_{\text{full}} }}$ values by pointwise substituting the $Q_{{i_{\text{full}} }}$ values. Equation (6) shows the subtraction step.

6 $$\Delta p_{{i_{\text{stent}} }} = \Delta p_{{i_{\text{full}} }} - (a_{\text{empty}} Q_{{i_{\text{full}} }}^{2} + b_{\text{empty}} Q_{{i_{\text{full}} }} )$$

The necessity of this step was to gather all the points {$\Delta p_{{i_{\text{stent}} }}$; $Q_{{i_{\text{full}} }}$} of the four measurements to fit a polynomial curve, where the confidence intervals of the coefficients could be calculated. Finally, the pressure drop of an FD stent in a given scenario can be characterized as follows:

7 $$\Delta p_{\text{stent}} = \left( {a \pm \delta a} \right)_{\text{stent}} Q^{2} + \left( {b \pm \delta b} \right)_{\text{stent}} Q$$

**Figure 5:**
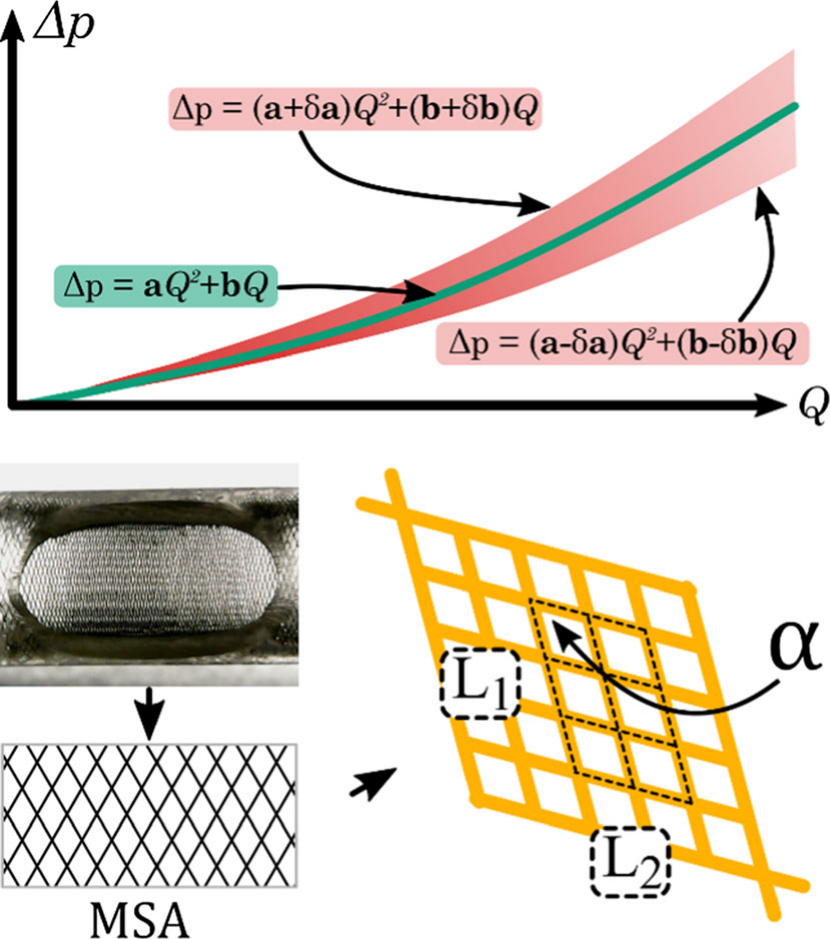
Illustration of the hydrodynamic resistance measurements. The thick green curve depicts the mean $\Delta p_{\text{stent}} \left( Q \right)$ curve, while the red shaded area is formed by coefficients with their upper and lower confidence bounds. Bottom: The methodology used for the MSA and pore density measurements.

In this equation *a*_stent_ and *b*_stent_ are the quadratic and linear coefficients and *δa*, *δb* are the corresponding intervals computed at 95% significance level leading to confidence bands around the mean curve. The graphical illustration of Eq. (7) can be seen in Fig. 5.

#### Metallic Surface Area and Pore Density

In addition, a relevant question is the relationship between the hydrodynamic resistance and the geometrical features of a given implantation scenario, namely the metallic surface area (MSA) and the pore density. To quantify these, during the measurements with the implanted devices a picture was taken before each measurement with a USB microscope to capture the disposition of the strut structure at the elliptical opening. In Fig. 5 the bottom left hand side panel depicts a picture taken with the microscope. To exclude cylindrical distortion, a rectangular window from the middle of the image was cut for post-processing. Afterwards, the contrast level was adjusted to fully distinguish between the background (white pixels) and the stent struts (black pixels). Then the MSA or metal coverage can be computed as follows:

8 $${\text{MSA}} = \frac{\text{Strut pixels}}{\text{All pixels}}$$

Subsequently, we measured the pore density with the same definition as.^23^ Using the depictions at the bottom right of Fig. 5 the pore density (PD) can be calculated.

9 $$\begin{aligned} {\text{Surface area}} = L_{1} L_{2} { \sin }\left( \alpha \right) \hfill \\ {\text{PD}} = \frac{{N_{\text{pore}} }}{\text{Surface area}} \left[ {\frac{\text{pore}}{{{\text{mm}}^{2} }}} \right] \hfill \\ \end{aligned}$$

where the value of *N*_pore_ represents the number of pores inside the rectangle covered by the Surface Area. An open source image processing software (ImageJ) was used to count the pixels and to measure the quantities for pore density estimation.

## Results

So far the paper has focused on measurement device development and the evaluation method. The following section will discuss the results of two stent placement scenarios with two types of FD stents (FD **A** and FD **B**). Two stents of 4 and 5 mm nominal diameter were deployed into holder tubes of the same diameter.

### Length Measurements

Table 3 summarizes the geometrical data of all the scenarios: the nominal diameter (*D*) and length (*L*_N_) from the product sheets, the deployed (*L*_D_) and unconstrained (*L*_U_) lengths from the measurements. Here the nominal and deployed diameters are identical, since the former is given by the manufacturer and the latter is the size of the holder tube. The unconstrained diameters of the FD **A** and FD **B** stents are respectively 0.25 and 0.3 mm larger than their nominal diameter. Compared to their nominal lengths, the deployed lengths become shorter in all scenarios for both types of FDs. Both types of FDs display further foreshortening in the unconstrained condition (with almost the same diameter). The deployed length of FD **A** stents remains close to their unconstrained length, while the elongation of deployed FD **B** type stents is significant compared to the unconstrained condition. Before going further, we remark that since the primary purpose of this paper is to demonstrate the measurement and the evaluation method, the reported results herein are valid for these particular deployment scenarios.

**Table 3:** Length measurement data.

Type	*D*	*L*_*N*_	*L*_*D*_	*L*_*U*_	*N*_*s*_
Unit	[mm]	[mm]	[mm]	[mm]	[–]
FD A	5	20	16,9	13,4	48
FD B	5	24	19,9	8,2	64
FD A	4	20	14,2	15,8	48
FD B	4	24	15,3	10,3	64

*L*_N_ and *D* are the nominal length and diameter from the manufacturers. *L*_D_ and *L*_U_ are the deployed and unconstrained lengths of the devices. N_s_ is the number of struts in the woven stent

### MSA and Pore Density Measurements

**Figure 6:**
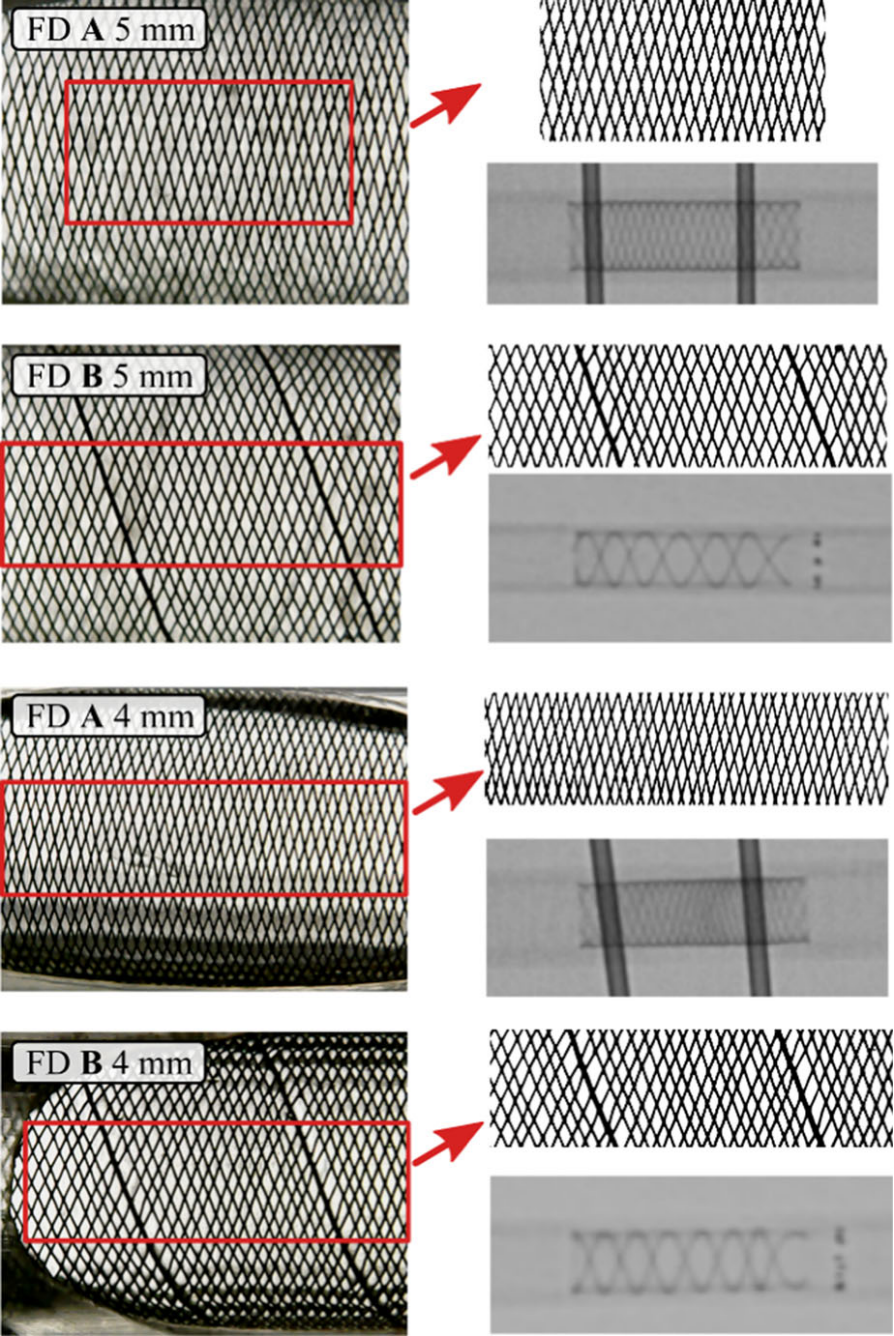
Microscopic pictures of the implanted stents. The red rectangle shows the window which was used for measuring the MSA. On the bottom right of each assembly an X-ray image is displayed after the deployment.

Figure 6 showcases the microscopic images, the captured and enhanced image windows and sample X-ray images for all the demonstrated cases. Table 4 contains the mean values of MSA and pore density with their respective standard deviations calculated for the four measurements.

**Table 4:** Mean values and their standard deviations of MSA and pore density, respectively for the deployed stents.

Type	MSA	SD	PD	SD
	[–]	[–]	[p/mm^2^]	[p/mm^2^]
FD A 5 mm	0.43	0.049	20.1	4.4
FD B 5 mm	0.39	0.021	28.7	4.9
FD A 4 mm	0.36	0.007	18.9	1.4
FD B 4 mm	0.45	0.026	28.6	6.7

In our current case, the MSA for FD **A** type displayed a somewhat higher mean value with higher deviation than the FD **B** type of the same diameter. Reversely, the pore density was approximately 40% higher for FD **B** (28.7 p/mm^2^) than for FD **A** (20.1 p/mm^2^), with both having almost the same standard deviation.

It can be seen in Table 4 that the MSA for the 4 mm diameter devices demonstrated a reversed order compared to the 5 mm ones. The 4 mm FD **B** type displayed higher MSA and pore density values than the FD **A** types. Although the SD of the quantities for FD **B** are much higher, not even the deviation bands overlap. For both stent types under unconstrained condition the MSA value was independent of the nominal diameter, **≈ **0.4 for FD **A** and **≈ **0.6 for FD **B**. While the pore density for both diameters of FD **B** changed significantly by deployment (from above 60 p/mm^2^ unconstrained to the values displayed in Table 4 deployed), it remained almost unchanged for FD **A** type stents.

### Hydrodynamic resistance measurements

**Figure 7:**
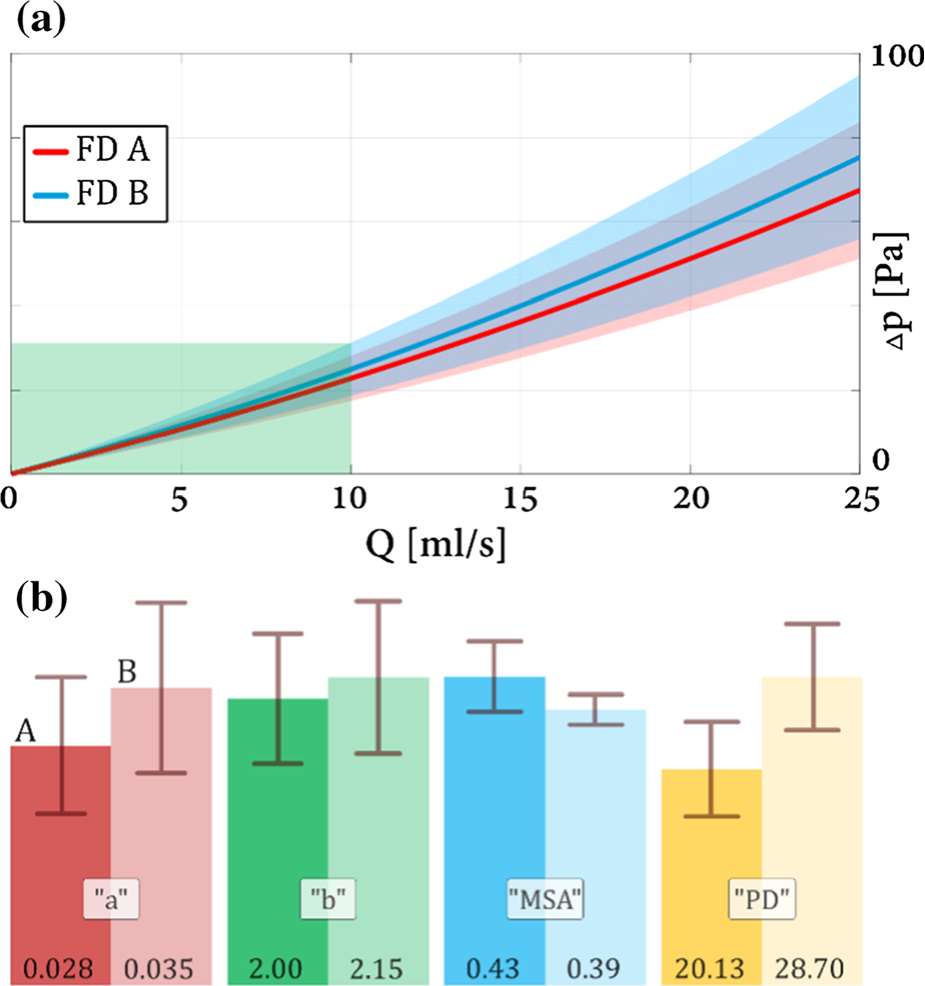
Top panel (a): $\Delta p_{\text{stent}} \left( Q \right)$ curves for the 5 mm nominal diameter FD devices. Bottom panel (b): linear and quadratic coefficients with their respective MSA and pore density values.

**Figure 8:**
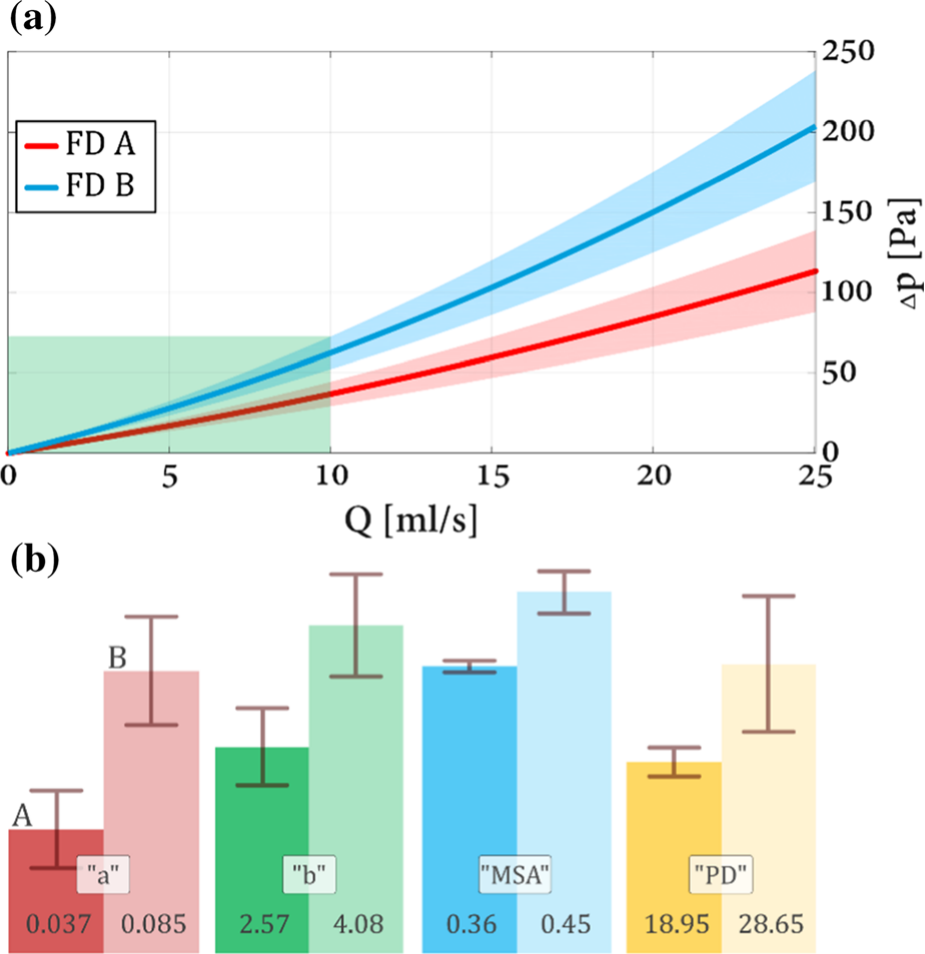
Top panel (a): $\Delta p_{\text{stent}} \left( Q \right)$ curves for the 4 mm nominal diameter FD devices. Bottom panel (b): linear and quadratic coefficients with their respective MSA and pore density values.

Turning now to the HR results, Figs. 7 and 8 depict the $\Delta p_{\text{stent}} \left( Q \right)$ curves for the 5 and 4 mm diameter stents, respectively. In these figures under the diagrams a bar graph can be observed (denoted by”(b)” in the figures) comprising all the geometrical and hydrodynamic indices of the two compared deployment scenarios. The coefficients and their confidence intervals from Figs. 7 and 8 are summarized in Table 5. The results in Table 5 tell us that the measurement accuracy was similar in all cases, as the confidence intervals relative to their respective mean values were all around 20–30%. This can be accounted for by small unperceivable movements of the stent during the measurements due to higher flow rates at the first few measurement points on the $\Delta p_{\text{stent}} \left( Q \right)$ curve. The average to peak flow rates in the internal carotid arteries are usually around 5–10 ml/s (Ford *et al*.^19^). To capture this in the figure, a green shaded rectangle shows the physiological region. Although in this particular region—in most of the cases—a linear relationship would be sufficient, since the peri-aneurismal flow field is rather complex (inflow jets, flow separations due to vessel curvature), possible higher local flow velocities necessitate the full quadratic relationship.

**Table 5:** Coefficients of $\Delta p_{\text{stent}} \left( Q \right)$ curves.

Type	a	δa	b	δb
FD A 5 mm	0.028	0.008	2.00	0.454
FD B 5 mm	0.035	0.01	2.15	0.533
FD A 4 mm	0.037	0.011	2.57	0.482
FD B 4 mm	0.0845	0.016	4.08	0.635

*a* and *δa* is the quadratic, *b* and *δb* is the linear coefficient with their respective confidence interval

As shown in Fig. 7 for the 5 mm devices, the pressure drop on FD **A** is lower than on FD **B**. Both the linear (“*b*”) and quadratic (“*a*”) coefficients are lower. The diagram also shows a wide range of overlap in the confidence bands. Surprisingly, the deployed FD **A** has a higher MSA value coupled with a lower resistance. On the other hand, FD **B** has a higher pore density coupled with higher resistance. In the physiological region up to 5 ml/s the two mean curves are within the line thickness, which in this flow rate regime implies the same effect on the peri-aneurismal flow fields.

Figure 8 depicts the same measurements on the 4 mm devices demonstrating substantial differences from the 5 mm scenarios. Similarly to the 5 mm devices, the 4 mm FD **B** type stent has a higher hydrodynamic resistance compared to FD **A**, but the difference here is much larger. The confidence bands do not overlap, not even in the physiological region. Looking at Table 5 we can see that both coefficients of the FD **B** are significantly higher, being almost twice as large as those of the FD **A**. The apparent inconsistencies of the data will be discussed below.

## Discussion

As pointed out in the Introduction, one of the aims of this paper is to associate the geometrical properties (MSA, pore density) with the HR, namely with the linear and quadratic coefficients of the resulting $\Delta p_{\text{stent}} \left( Q \right)$ curve of any given deployment scenario. In order to understand the relationship between these characteristics, we have to know all the information about the given deployment scenario, since hardly noticeable changes in the length and homogeneity of the strut structure can lead to considerably different results. Another source of measurement uncertainty that needed to be addressed came from the specific design of the measurement rig. We remind the reader that the system measures the hydrodynamic resistance for *perpendicular* flow through the stent, accommodating to the conditions of the Darcy–Forcheimer law. At a later stage it will be possible to measure also other angles of attack.

Only one previous study^21^ tried to investigate the relationship between these properties experimentally but a highly influential parameter was not taken into consideration, the length of the deployment. This parameter affects the MSA and PD values (and thus the hydrodynamic resistance), since the change in the length of the stent is in a mechanical relationship with the resulting strut structure. Nevertheless, their conclusion that a single parameter as MSA or PD is not sufficient to describe the hydrodynamic resistance characteristics of a stent is in agreement with our findings. Another *in vivo* study^25^ discussed that the MSA of a stent type depends on its nominal diameter, yet, they also ignore the effect of the deployment length.

Now we turn to the interpretation of our results. The FD **A** stent has 48 struts over a nominal length of 20 mm, while FD **B** has 64 struts over 24 mm. This means that FD **B** has a higher strut density. Common sense dictates that a higher density corresponds to a higher MSA and also to a higher resistance. In the 4 mm case the results correspond to our expectations. The difference between the HRs of the two stents is even larger than would be normally expected due to the very different deployment lengths: *L*_D_/*L*_U_ are 0.9 and 1.48 for FD **A** and FD **B**, respectively. In the 5 mm cases, however, the results are highly surprising. Lower MSA is coupled with higher HR and higher pore density. Let us notice first that the difference between the MSA values is small and among the other variables it is not too large, either. This is reflected in Fig. 7 where the confidence bands of the curves bite into each other. This result can be explained as follows. In the unconstrained condition the struts of both stents form squeezed rhombi. If the stent is elongated, the rhombi approach squares, leading to lower MSA. Since in our current 5 mm case the ratio *L*_D_/*L*_U_ was 1.26 and 2.42 for FD **A** and FD **B**, respectively, this explains the higher MSA for FD **A**. At the same time, because of the higher pore density and close to equal MSA the resistance of FD **B** is still higher.

Since here only a few measurement results have been presented, a general hypothesis for the relationship between the geometrical and hydrodynamic parameters cannot be set up. However, some qualitative remarks can be given. In general, the deployment length ratio of FD **B** type stents is higher than that of FD **A** ones in those scenarios where the FDs are implanted into their respective nominal size holder tubes. Despite the fact that FD **B** type stents have 64 struts, it is possible for them to have a lower MSA than the FD **A** type of stents in certain deployment scenarios and still have higher hydrodynamic impact, since the higher number of struts can produce a higher pore density.

## Conclusions

This study described the development of an experimental rig and a reliable protocol for the hydrodynamic resistance measurements of intracranial flow diverter stents used in the endovascular therapy of brain aneurysms. Some stages of the development of the measurement system together with the associated difficulties were discussed. The measurement system in its present state works reliably and provides useful information about the HR and thus an important element of the feasibility of FD stents. The deployment scenarios were analysed by means of MSA, pore density and deployment length. Although the current study is based on a few results only, the findings suggest that the hemodynamic impact of a flow diverter deployment scenario is determined by the length, MSA and pore density collectively. Furthermore, the unconstrained geometric properties can serve as a base for understanding the behaviour of certain stent types. The observations in this paper provide a good starting point for an ongoing study that incorporates more deployment scenarios to quantitatively understand the relationship between the geometric and hydrodynamic properties based on our measurements and further CFD simulations.
